# Augmented Cooper test: Biomechanical contributions to endurance performance

**DOI:** 10.3389/fspor.2022.935272

**Published:** 2022-09-14

**Authors:** Salil Apte, Simone Troxler, Cyril Besson, Vincent Gremeaux, Kamiar Aminian

**Affiliations:** ^1^Laboratory of Movement Analysis and Measurement, École Polytechnique Fédérale de Lausanne (EPFL), Lausanne, Switzerland; ^2^Sport Medicine Unit, Division of Physical Medicine and Rehabilitation, Swiss Olympic Medical Center, Lausanne University Hospital, Lausanne, Switzerland; ^3^Institute of Sport Sciences, University of Lausanne, Lausanne, Switzerland

**Keywords:** wearable sensors, biomechanical profile, acute fatigue, continuous assessment, running distance

## Abstract

Running mechanics are modifiable with training and adopting an economical running technique can improve running economy and hence performance. While field measurement of running economy is cumbersome, running mechanics can be assessed accurately and conveniently using wearable inertial measurement units (IMUs). In this work, we extended this wearables-based approach to the Cooper test, by assessing the relative contribution of running biomechanics to the endurance performance. Furthermore, we explored different methods of estimating the distance covered in the Cooper test using a wearable global navigation satellite system (GNSS) receiver. Thirty-three runners (18 highly trained and 15 recreational) performed an incremental laboratory treadmill test to measure their maximum aerobic speed (MAS) and speed at the second ventilatory threshold (sVT2). They completed a 12-minute Cooper running test with foot-worm IMUs and a chest-worn GNSS-IMU on a running track 1–2 weeks later. Using the GNSS receiver, an accurate estimation of the 12-minute distance was obtained (accuracy of 16.5 m and precision of 1.1%). Using this distance, we showed a reliable estimation [R^2^ > 0.9, RMSE ϵ (0.07, 0.25) km/h] of the MAS and sVT2. Biomechanical metrics were extracted using validated algorithm and their association with endurance performance was estimated. Additionally, the high-/low-performance runners were compared using pairwise statistical testing. All performance variables, MAS, sVT2, and average speed during Cooper test, were predicted with an acceptable error (R^2^ ≥ 0.65, RMSE ≤ 1.80 kmh^−1^) using only the biomechanical metrics. The most relevant metrics were used to develop a biomechanical profile representing the running technique and its temporal evolution with acute fatigue, identifying different profiles for runners with highest and lowest endurance performance. This profile could potentially be used in standardized functional capacity measurements to improve personalization of training and rehabilitation programs.

## Introduction

Training prescription for runners is typically based on personal physiological capacity (Reilly et al., 2009), with training intensity determined by a certain fraction of variables such as maximal oxygen uptake (VO_2max_), maximal heart rate (HR_max_), or others, usually assessed during exercise with increasing intensity (Nes et al., 2013). Both physiological variables are indicators of cardiorespiratory capacity (Seiler, 2011). However, given the difficulty in measuring these variables in field training sessions, other metrics may be more convenient to use. For example, the maximal aerobic speed (MAS), i.e., running speed when VO_2max_ is reached, is commonly used to prescribe training intensity (Berthoin et al., 1994). Another approach for prescription of training intensity is to use zones near the ventilatory threshold (VT) and/or lactate threshold (LT) because they represent the submaximal response of the individual athletes and indicate their ability to sustain a high fraction of VO_2max_ for an extended period of time (Bassett, 2000). Athletes exhibit different levels of lactate accumulation for the same fraction of VO_2max_, so using thresholds instead of VO_2max_ may produce less interindividual variation in the metabolic response and create a more homogeneous training stimulus (Mann et al., 2013). An important reason for using VT is polarized endurance training (PET), which is based on a training that is mostly below the first VT (VT1) and10–20% being at/and above the second VT (VT2) (Muñoz et al., 2014). PET may increase positive adaptation to training stimuli and reduce the risk of overtraining, chronic fatigue, and injury (Muñoz et al., 2014; Wolpern et al., 2015). Evidence shows that elite endurance athletes perform their training mainly below VT1/LT1 and/or clearly above the VT2/LT2, thus highlighting the importance of these thresholds in training (Haugen et al., 2022).

The gold standard for measuring VO_2max_ and VT2, and consequently the MAS and speed at VT2 (sVT2) is a treadmill test in the laboratory with gas exchange analysis (Bellenger et al., 2015). However, such a test requires highly trained personnel, is expensive, and only one person can be tested at a time. To overcome these constraints, it seems attractive to develop and conduct simple field tests that do not require extensive equipment, are inexpensive and can be integrated into athletes' routines. In these tests, measurement accuracy is partially sacrificed in favor of ease of use and potential for repeatability throughout the season for multiple athletes simultaneously. An example is the Cooper field test (Cooper, 1968), which is used to estimate VO_2max_ based on the total distance run. It is a simple test that involves 12 mins of track running with self-paced maximal effort and provides a good assessment of VO_2max_, MAS, and a reasonable prediction of half marathon time (Alvero-Cruz et al., 2019). Although incremental treadmill testing has been used to predict VT using portable near-infrared spectroscopy (NIRS) (Rodrigo-Carranza et al., 2021) or portable heart rate monitor (Gronwald et al., 2020), to our knowledge there is currently no simple field test for predicting sVT2.

The performance of long-distance runners depends not only on the VO_2max_ and the ability to maintain a high fraction of VO_2max_ during running but also on running economy (RE) (Moore, 2016; Folland et al., 2017; Preece et al., 2019). RE is the metabolic energy expenditure for a given speed during submaximal running and can vary by up to 30% among runners with a similar VO_2max_ (Daniels, 1985; Morgan et al., 1989). Running mechanics determine the mechanical power and propulsion produced for a given energy expenditure, thus influencing RE. Running biomechanics during ground contact, particularly during the propulsive phase, show a strong correlation with RE during treadmill running (Saunders et al., 2004; Beattie et al., 2014; Moore, 2016). Measuring RE during field running requires the use of a portable gas analyzer, which is expensive and impractical, whereas field running biomechanics can be accurately and conveniently assessed using wearable inertial measurement units (IMUs) (Strohrmann et al., 2012; Buckley et al., 2017; Benson et al., 2018). The use of an economical running technique can improve RE and thus performance (Saunders et al., 2004; Moore, 2016). Therefore, evaluating running biomechanics during a field capacity test could greatly improve endurance performance information and help identify the biomechanical factors that contribute to endurance performance.

Research in this direction has mainly focused on differentiating between highly experienced and inexperienced runners based on their running technique. Clermont et al. and Carter et al. used data from IMU and collected using fixed-speed treadmill protocols (Clermont et al., 2019b; Carter et al., 2022). Preece et al. extended this approach to run overground over a distance of 32 m at four different fixed speeds and analyzed the running kinetics and kinematics at three different steps during the run (Preece et al., 2019). While these studies showed promising results and highlighted important biomechanical characteristics of high-performance runners, they did not account for the natural variability (Meardon et al., 2011; Mo and Chow, 2018) and asymmetry (Radzak et al., 2017; Beck et al., 2018) that occur at self-selected speeds, nor did they consider the effects of fatigue when running longer distances (Prigent et al., 2022), which are common in field tests of endurance capacity. The use of wearable IMU and global navigation satellite systems (GNSS) has shown promise in the improvement and augmentation of field testing for countermovement jump (Picerno et al., 2011), single-leg hop (Ahmadian et al., 2020), sprint (Apte et al., 2020), balance (Johnston et al., 2016), and so on. In this study, we aim to extend this wearables-based approach to the Cooper test by evaluating the relative contribution of running biomechanics to the endurance performance. In addition, we investigate whether the use of biomechanical parameters improves the prediction of MAS and sVT2 during the field test and explore different methods for estimating the distance covered in the Cooper test using a wearable GNSS receiver.

## Materials and equipment

### Participants and study design

We conducted measurements with 18 highly trained (18 males, age 27.7 ± 5.4 years; height 178.8 ± 4.8 cm; weight 69.6 ± 10.1 kg; personal best below 90 mins for a half-marathon) and 15 recreational runners (5 females, 10 males, age 31.5 ± 5.9 years; height 173.7 ± 9.9 cm; weight 67.8 ± 14.7 kg), all runners aged between 18 and 50 years. To recruit highly trained runners, if there was no time reference in this distance, we classified the participants based on their personal best on 10 km or 5 km with the Riegel Formula's half marathon time estimation (23). The university human research ethics committee (HREC 053-2020) approved the study and all participants provided written consent before the data collection. Participants performed an incremental laboratory treadmill test to measure MAS and sVT2. After 1–2 weeks, they completed a Cooper running test with wearable sensors on a running track.

### Laboratory test

**Figure 1:**
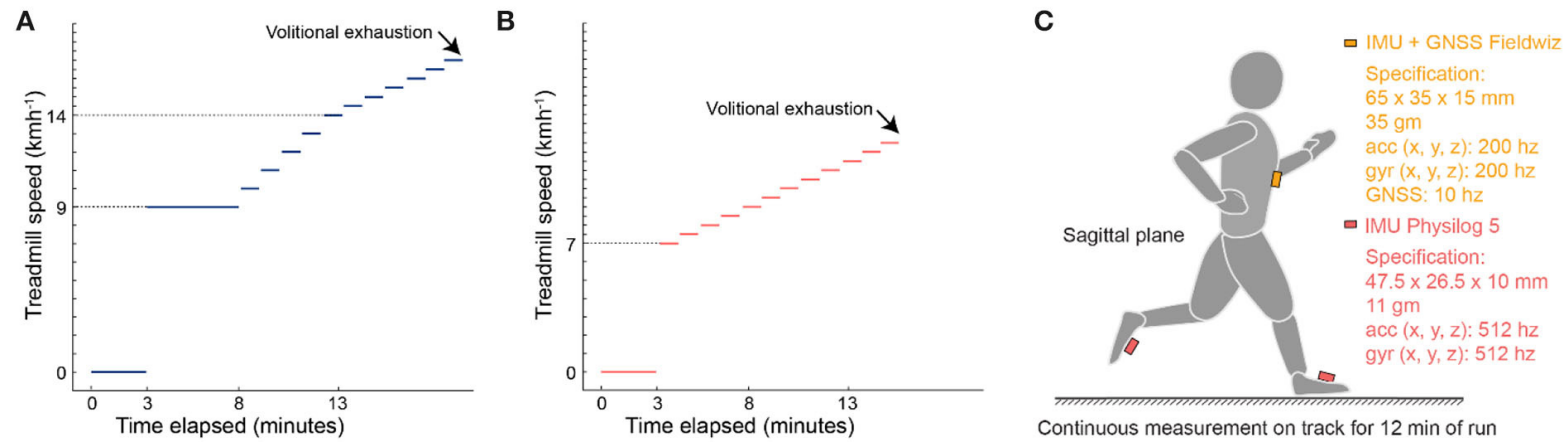
Protocol and sensor setup. **(A)** Incremental speed protocol till volitional exhaustion for highly experienced runners. **(B)** Incremental speed protocol till volitional exhaustion for amateur runners. **(C)** Sensor configuration for field measurement. IMU, inertial measurement unit; GNSS, global navigation satellite system; acc, accelerometer; gyr, gyroscope.

Prior to the lab test, participants were instructed to have no meals 2 h before the test, and not have performed intense training 48 h prior to the test. Height and weight of the participants were measured before they performed a maximal incremental running test on a treadmill (Pulsar, HP Cosmos, Nussdorf-Traunstein, Germany), while wearing a mask for Cortex Metalyzer 3B gas exchange analyzer (Cortex Biophysik GmbH, Leipzig, Germany) and a heart rate belt (H10, Polar Electro OY, Kempele, Finland) on the chest. For the highly trained group (Figure 1A), the testing protocol involved 3 mins of rest, a 5-min warm-up at 9 kmh^−1^, followed by an increase in the speed of 1 kmh^−1^ every minute until 14 kmh^−1^, and finally an increment of 0.5 kmh^−1^ every minute until volitional exhaustion. For the second group (Figure 1B), the protocol involved a 7 kmh^−1^ start, followed by increments of 0.5 kmh^−1^. Oxygen consumption (VO_2_), carbon dioxide production (VCO_2_), ventilation (VE), and heart rate (HR) were measured continuously throughout the test. Participants were provided encouragement throughout the test to ensure attainment of maximal effort.

Maximal effort was controlled according to the following criteria: plateauing of the VO_2_-speed relationship with VO_2_ increasing by <2 ml·kg^−1^·min^−1^ despite speed increase, a peak respiratory exchange ratio (RER) >1.10, or peak HR within 10 beats min^−1^ of the age-predicted maximum. Gas exchange variables were averaged on 20 s. The speed value at which the VO_2_ plateau began was considered as MAS. Second VT (VT2) was determined according to 3 criteria (Beaver et al., 1986; Cerezuela-Espejo et al., 2018) by an experienced exercise physiologist: (1) increase in both respiratory equivalent (VE/VO_2_ and VE/VCO_2_), (2) a decrease in PETCO2, and (3) a loss of linearity from VE/VCO_2_ plots. The speed attained at VT2 was considered as sVT2.

### Field test

**Figure 2:**
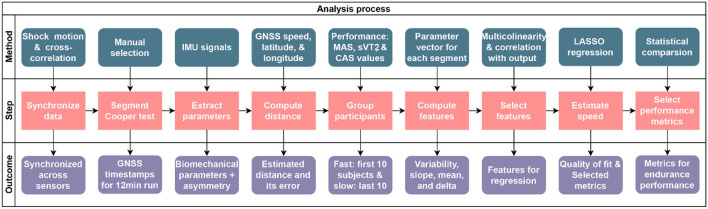
Flowchart of the overall procedure for extraction and selection of metrics. LASSO, least absolute shrinkage and selection operator; CAS, average speed during the 12-minute Cooper test.

After 10 mins of warm-up, participants were equipped with an IMU (Physilog 5, Gaitup SA, Switzerland) on each foot and a GNSS-IMU sensor (Fieldwiz, ASI, Switzerland) on the chest using a belt with electrodes (Polar Pro Strap, Polar Electro Oy, Finland). Apart from the sensor setup (Figure 2C), the participants dressed as they would for an endurance running race. The Fieldwiz and Physilog 5 wearable sensors were chosen because they have already been used successfully for continuous analysis of running in the field and do not hinder the running movement (Prigent et al., 2022). Fieldwiz was used with a sampling frequency of 200 Hz for the IMU, 250 Hz for the ECG, and 10 Hz for the GNSS receiver. The ECG was not utilized as the focus of this study was on biomechanical contributions to endurance performance. The Physilog 5 IMU was sampled at 512 Hz, with a range of ±16 g for the accelerometer and ±2,000 deg/s for the gyroscope. The participants ran on a 400 m tartan track for 12 minutes and were instructed to cover highest distance possible. They were asked to rate their perceived fatigue from 1 to 10 before/after the run using the rating of fatigue (ROF) scale (Micklewright et al., 2017), which considers 1 as no fatigue and 10 as maximal. The participants performed the test in groups of 2–4 to increase their motivation. Two instructors provided verbal encouragement, supervised the test, and calculated the total distance covered in 12 mins by counting the number of 400 m laps and the meters covered in the final lap. The distance (D_ref_) was measured by considering the closest scale on the track, which provides a resolution of 10 m and are usually used to measure distance during training.

## Methods

**Figure 3:**
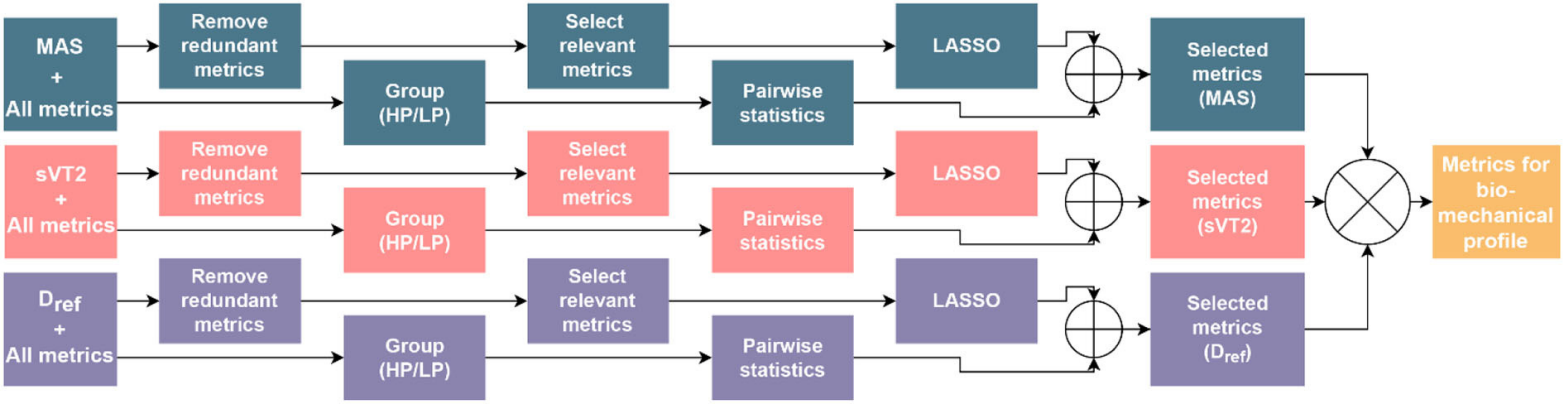
Procedure for selection of performance metrics for the biomechanical profile.

**Figure 4:**
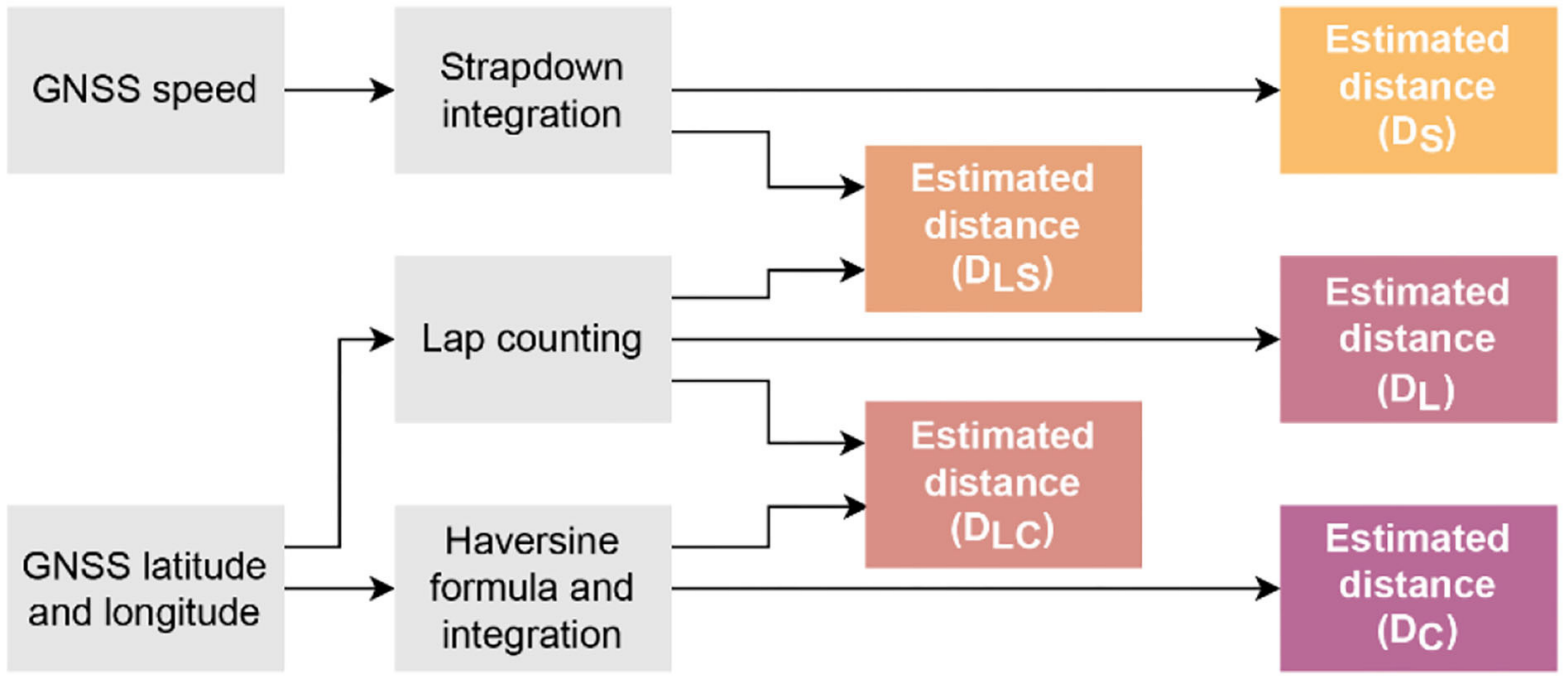
Different methods for the estimation of distance covered over the 12-min run.

The flowchart of the overall procedure for the pre-processing, parameter estimation, and extraction and selection of metrics is presented in Figure 2, and detailed explanations are provided in the sections below. In addition, Figure 3 provides detailed information about the selection of metrics and Figure 4 about the distance estimation.

### Preprocessing and parameter estimation

The pre-processing steps include synchronization of the sensors and segmentation of the Cooper test run (Figure 2) for each participant. To synchronize the Fieldwiz and Physilog 5 sensors, we performed a shock movement, before and after the 12-min run. This movement consists of a quick up and down movement on the vertical axis while holding all sensors together (Caruso et al., 2019). Since the same acceleration data were recorded on both sensors, we computed the lag between their acceleration signals with cross-correlation and used this lag to adjust their timestamps. Segmentation of data for each participant was done on the basis of the magnitude of acceleration norm from the IMU on the right foot, the ground speed data from GNSS and the known duration of 12 mins.

We removed outliers that were more than two standard deviations away from the mean value over a 1-min sliding window from the GNSS ground speed signal and replaced them with linearly interpolated values. The 12-min run was segmented into individual gait cycles using the angular velocity values of the right foot at mid-swings, following a validated algorithm (Falbriard et al., 2018). For each gait cycle, we estimated the gait temporal parameters like contact time (CT), flight time (FT), swing time (ST), and gait cycle time (GT), and kinematics parameters such as peak swing velocity of the foot (PSV), foot strike angle in sagittal plane (FSA), and foot eversion angle (FEA) at initial contact (Falbriard et al., 2018, 2020). Duty factor (DF) is one of the main descriptors of running style, which we estimated as the percentage ratio of CT to GT (Alexander, 1991) for every gait cycle. Using the spring-mass model gait model (Morin et al., 2005), we computed the vertical stiffness (VS) due its importance for efficient storage and return of elastic energy (da Rosa et al., 2019). Meyer et al. have presented the computation of the above-mentioned parameters in detail (Meyer et al., 2021). Fatigue has an effect on asymmetry of gait spatiotemporal parameters, and thus to understand its influence on endurance performance, we quantified the asymmetry using the symmetry index (SI):


(1) $$\begin{array}{r} SI=\;2\;x\frac{\left|X_{L}-X_{R}\right|}{\left(X_{L}+X_{R}\right)}x\;100\% \end{array}$$

where *X*_*R*_ and *X*_*L*_ are parameters for the right and left limbs. We first computed SI for the gait cycle time to check the validity of the SI, as the cycle time should present an SI close to zero. Following that, we used SI (Figure 1) with four gait parameters, CT (CT_SI_), flight time (FT_SI_), swing time (ST_SI_), and PSV (PSV_SI_), based on their evolution with acute fatigue during endurance running (Apte et al., 2021; Prigent et al., 2022). All the computations were done using MATLAB R2020b, and the plots showing the evolution of biomechanical parameters and running speed during the Cooper test were created using the Gramm package (Morel, 2018) and smoothing (Eilers, 2003) for averaging the trajectories.

### Extraction of metrics

To address the influence of accelerating at the beginning of the test and strategy of exerting higher near the end of the test, we removed the first and last minute of the data from subsequent analysis. Within those 10 mins, for each biomechanical parameter, we considered five different *time segments* (Table 1) for extraction of metrics:

**Table 1:** List of biomechanical parameters (units) extracted using the data from foot IMU sensors, the features computed on these parameters, and the time segments over which they are computed.

Biomechanical parameters	1. Contact time (CT) (ms), 2. Flight time (FT) (ms), 3. Swing time (ST) (ms), 4. Gait cycle time (GT) (ms), 5. Vertical stiffness (VS) (kNm-1), 6. Foot strike angle (FSA) (°), 7. Foot eversion angle (FEA) (°), 8. Peak swing velocity (PSV) (°s-1), 9. Duty factor (DF) (%) 10. CT asymmetry (CTSI) (%) 11. FT asymmetry (FTSI) (%) 12. ST asymmetry (STSI) (%) 13. PSV asymmetry (PSVSI) (%)
Features	1. Mean (μ), 2. Variability (σ)—not for asymmetry parameters, 3. Slope (m)
Time segments	1. Total (t): Minute 2nd to 11th, 2. Steady (sy): Minute 5th to 8th, 3. Start (s): 2nd minute, 4. End (e): 11th minute, 5. Delta (d): 11th minute-−2nd minute
Metric example	Mean feature of vertical stiffness for total time segment: μVSt

An example notation for one metric is provided in the last row.

- Total (t): all 10 mins.
- Steady (sy): running at the middle (Minute 5th to 8th) of the test.
- Start (s): first minute of the remaining 10 mins.
- End (e): last minute for the same.
- Delta (d): difference between the parameter values for the start and end segments.

For all the time segments, three features were extracted—mean (μ): arithmetic mean of parameter values over one time segment; variability (σ): standard deviation of parameter values over a window of 10 gait cycles and the arithmetic mean of these windows over a time segment; and slope (m): ratio of the difference between the last and the first parameter values of a time segment and the length of the time segment. Mean (μ) and slope (m) features were computed for all biomechanical parameters, whereas variability (σ) only for the first nine parameters and not asymmetry parameters. Following this method, we obtained a total of 175 metrics using 13 biomechanical parameters, five segments of time, and three features. For example, μVSt denotes “Mean feature (μ) of vertical stiffness (VS) for Total time segment (t).” For each parameter (except asymmetry), we computed one value per gait cycle for the left and right foots but used only the information from the right foot for the extraction of metrics.

#### Categorization

In addition to physiological aspects, performance during endurance running depends on the RE, the ability of runners to efficiently translate metabolic energy into mechanical work, and the capacity to sustain an efficient running technique over a relatively long duration (Folland et al., 2017; Moore et al., 2019; Preece et al., 2019). Based on these findings, we divided the above-mentioned 175 metrics into five different categories, with the goal of understanding the relative contribution of each category to the endurance performance:

1. *Technique*: It is a set of metrics that describe the running technique. Higher VS (lower vertical oscillation) has been associated with better RE (Moore, 2016; Zhang et al., 2021), durations of CT and FT have been used to classify running styles (Gindre et al., 2015), FSA and FEA directly influence the direction and magnitude of impact force at first contact (Lieberman et al., 2010; Muniz-Pardos et al., 2018; Hoenig et al., 2020), and DF is considered an independent descriptor of running style (van Oeveren et al., 2021). Thus, we considered only the mean feature (μ) for CT, FT, VS, FSA, FEA, and DF for all time segments except Delta in this category.
2. *Regularity*: It is the category of metrics that quantify the variability of gait and include only the variability feature (σ) for all parameters except asymmetry, across all time segments except Delta. Variability of stride has a functional purpose, considered to offer flexibility of adaption to task and environmental constraints (Hausdorff, 2007). Stride time variability has been previously studied to investigate differences in trained and non-trained runners (Nakayama et al., 2010), and also to investigate the influence of acute fatigue (Gindre et al., 2015; Mo and Chow, 2018).
3. *Asymmetry*: As the name implies, this set of metrics quantify the asymmetry of gait cycles, using only the mean feature (μ) for CT_SI_, FT_SI_, St_SI_, and PSV_SI_, across all time segments except Delta. A 10% increase in CT_SI_ can lead to a 7.8% increase in the metabolic cost of running (Beck et al., 2018) and increasing asymmetry has been linked to overuse injuries due to increase in kinetic demands (Radzak et al., 2017).
4. *Fatigue*: Acute fatigue has an adverse effect on technique during prolonged running, by increasing the CT, DF, reducing FSA, VS, and so on (Apte et al., 2021; Meyer et al., 2021; Prigent et al., 2022). The ability to maintain an efficient running technique for a longer duration can thus improve the endurance performance. To quantify this ability, for all parameters, we used the μ, σ, and m features for Delta time segment and only the slope feature (m) for other segments.
5. *Pace*: We added another category for metrics that quantify the rate of movement and did not fit into the previous four categories. Although the gait cycle time (cadence) is not necessarily linked to efficiency of technique or fatigue resistance, it is often used for the performance evaluation and manipulation of running speed via different pacing strategies (Hausswirth and Brisswalter, 2008; Musgjerd et al., 2021). In addition to μ feature for GT, we also included the μ feature for ST and PSV for all time segments except Delta, in this group.

### Selection of metrics

To select the metrics that contribute to endurance performance, we considered three performance variables, the MAS and sVT2 obtained in the lab measurements, and the average speed during the Cooper test (CAS). Unlike the VO_2max_, it is convenient to prescribe and measure training intensity in terms of MAS and sVT2 due to the ease of measuring speed in the field. Use of CAS instead of total distance allows us to maintain the same units (kmh^−1^) and similar magnitude across the performance variables, thus enabling a reasonable comparison for the errors in their prediction. To streamline the number of metrics, we first normalized each metrics using z-score normalization across 33 participants and tested the normalized metrics for their Pearson correlation with each other. Within metric pairs showing a correlation coefficient above 0.95, the metric computed over a larger time segment was retained. Using this multicollinearity property (Mansfield and Helms, 1982), we reduced the number of metrics. To further reduce the metrics, their Pearson correlation coefficient (r) was computed in relation to MAS, sVT2, and CAS, and only the metrics with r ≥ 0.3 were retained for the final modeling step.

In the next step, to investigate the combined predictive power of the biomechanical metrics and D_ref_, we estimated the MAS and sVT2 using linear regression, once using D_ref_ and once with the D_ref_ and the biomechanical metrics selected in the previous steps. To understand the relative contribution of biomechanical metrics to endurance performance, we repeated the same process for MAS, sVT2, and D_ref_ with only the biomechanical metrics, using the least absolute shrinkage and selection operator (LASSO) method for metric selection (Hastie et al., 2001). This is a forward-looking selection for linear regression, which enables interpretability of the model, and can also enhance the prediction accuracy. Using leave-one-out-cross-validation with the LASSO method (Shao, 1993), we estimated the coefficients for each metric for predicting the three performance variables. Within the results of the LASSO method, we picked the coefficient vector with the least number of non-zero coefficients that led to an error of one standard deviation higher than that of the minimum mean-square error (Hastie et al., 2001). This led to a minimal model with a reasonable level of accuracy in prediction and reduced the chance of overfitting (Loh, 2011). Among the metrics with non-zero coefficients, we removed those with a relative weight of less than 5% of the total weights, due to their minimal importance. Furthermore, we summed the absolute weights of variables within the same category to quantify the relative contribution of each category to the regression model. The prediction results of all the regression processes are presented in terms of the cross-validated determination coefficient (R^2^) and the root-mean-square error (RMSE) in kmh^−1^. R^2^ determines the degree of association between predicted and actual performance variables, and the RMSE quantifies the difference between them. The overall process is illustrated in Figure 3.

Research has shown that non-linear shifts in gait parameters with the increase in speed possibly are related to a transition to a sprinting-like technique (Burns et al., 2021) at high speeds. To consider these non-linear transitions and to complement the selection of metrics through linear methods, we also conducted statistical analysis to investigate the differences between the 10 highest (HP) and 10 lowest (LP) performing participants according to MAS, sVT2, and D_ref_. The reason for considering all three factors separately is that the participants comprising HP and LP may differ depending on the performance variable under consideration. The metrics selected using multicollinearity were compared using a pairwise Welch's t-test with the statistical significance set at *p* < 0.05. This test was preferred over the Student's t-test due to unequal variances for the fast and slow groups (Ruxton, 2006). The effect size was calculated using the same formulation as Cohen's d (Gignac and Szodorai, 2016). For every performance variables, the metrics that were selected through LASSO and those with statistically significant differences were combined (union of sets). Following this, an intersection of these three (MAS, sVT2, and D_ref_) sets was used to select metrics that contribute mainly to the endurance performance and a visual profile representing these metrics and their respective categories was developed. As an illustration of its utility, we represented the five highest and five lowest performing participants according to their MAS and sVT2 on this profile.

### Distance estimation

Cooper test uses the total distance D_ref_ (in km) covered in 12 mins to estimate the VO_2max_ (ml kg^−1^ min^−1^) and MAS as follows (Léger and Mercier, 1984; Bandyopadhyay, 2015):


(2) $$\begin{array}{r} {\overset{∙}{V}O}_{2max}=22.351\times D_{ref}-\;11.288\\ MAS=\;\frac{{\overset{∙}{V}O}_{2max}}{3.5} \end{array}$$

Since the MAS estimation is directly dependent on the distance, it is important to estimate the distance accurately. The reference value for this distance (D_ref_) corresponds to the distance measured at the 10 m markers on the track. We used five different methods for estimating the distance (Figure 4) and compared them to the reference (D_ref_) using Bland-Altman analysis, mean absolute error (MAE), and percentage (Median ± IQR) error. We computed the percentage error for every method across all participants. Below is a brief description of each method:

1. The distance at the end of 12 minutes obtained from the strapdown integration of GNSS ground speed (without outliers) was considered the total distance (D_S_).
2. Using the Haversine formula (Robusto, 1957) with the latitude and longitude coordinates from the GNSS sensor, distance at the end of test was considered as total distance (D_C_).
3. The average distance (d_a_) between the peaks on the latitude signal was considered to be the time required to complete one lap. This was followed by estimating the number of laps by counting the number of peaks (n_p_) and length of signal (l_s_) outside the peaks was computed. Since the length of one lap is 400 m, the total distance was computed as:

(3) $$D_{L}=\;(n_{p}-1)\times 400+\;\frac{l_{s}}{d_{a}}\times 400$$

4. Combination of the first and third methods, by counting the number of laps using peak detection and using strapdown integration of ground speed on the signal outside the peaks. The total distance (D_LS_) is the sum of number of laps multiplied by 400 and the total distance on the strapdown integration before and after the first and last peaks, respectively.
5. Combination of the second and third methods, by counting the number of laps using peak detection and using Haversine formula with the coordinates on the signal outside the peaks. The total distance (D_LC_) is the sum of number of laps multiplied by 400 and the distance obtained with the coordinates before and after the first and last peaks, respectively.

## Results

**Figure 5:**
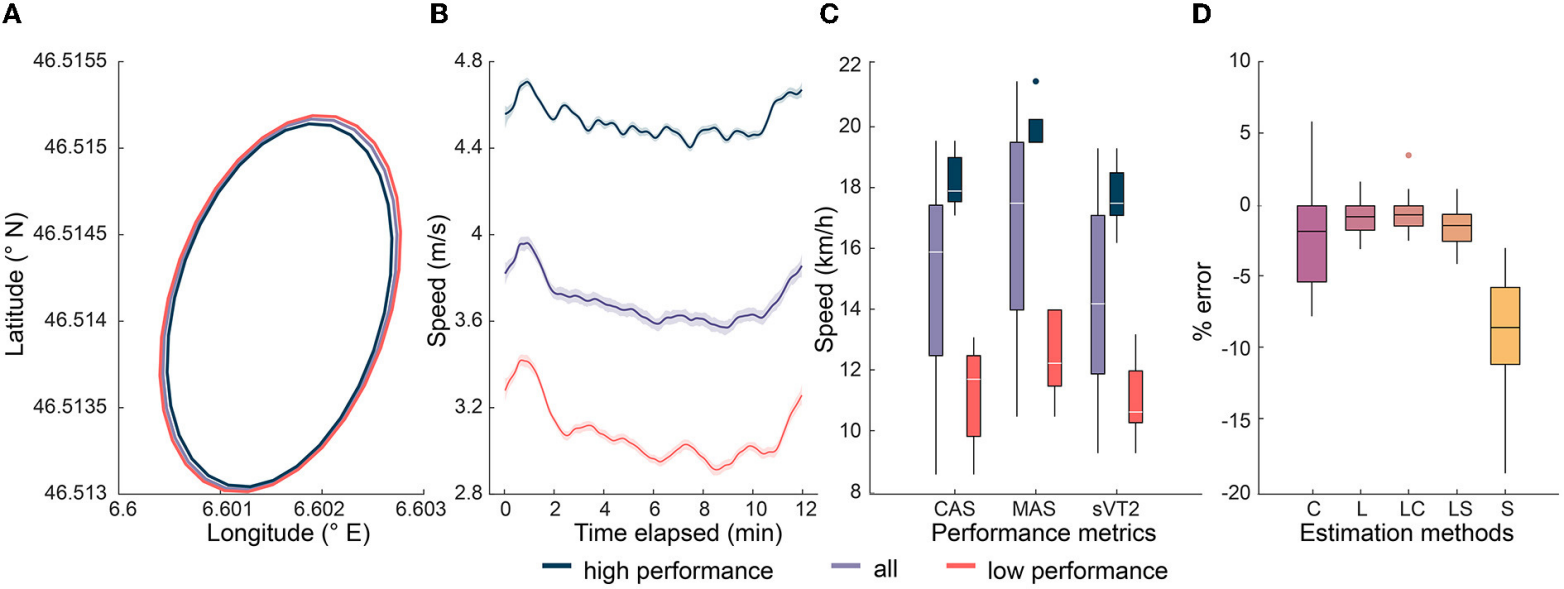
Performance of participants grouped according to D_ref_ and GNSS tracking. The smoothed mean of original profiles and the 95% confidence interval is shown for easier comprehension of their overall group trend and and plotted using the Gramm toolbox (Morel, 2018). **(A)** Representative trajectory of the run during the Cooper test. **(B)** Representative speed profile of the participants during the Cooper test. **(C)** Xoxplot showing the median and interquartile range of performance across three speed variables. **(D)** Median and IQR of error in the estimation of distance using five different methods, with C, L, and S corresponding to methods based on Haversine formula with the GNSS coordinates, lap counting, and strapdown integration of ground speed. LC and LS refer to a combination of lap counting with methods based on ground speed and coordinates respectively.

All 33 participants completed the 12 mins of Cooper test with a maximal effort, reporting an ROF ≥ 8 at the end. Representative trajectories are shown in Figure 5A for participants grouped according to D_ref_, with the latitude and longitudinal values aligning well with those of the track at the stadium. Participant's running speed (Figure 5B) generally decreased over 12 mins of Cooper test, except for the first and last minute, which showed an increase. As expected, the HP group showed higher mean speed and a lower reduction in speed with time. Figure 5C shows the performance of participants for the MAS, sVT2, and CAS, with the range of speeds being 9 kmh^−1^ to 21.5 kmh^−1^ and an average difference of around 7 kmh^−1^ between the top and bottom 10 participants for all three performance variables. However, the top 10 participants according to each variable were not the same. The details on their performance can be found in Supplementary Table S1.

### Distance and speed estimation

The distance estimated using all five methods showed a median error of −0.6 to −8.4%, with the strapdown integration of speed presenting the highest MAE (250 m) and the lap counting plus Haversine formula presenting the lowest (26.5 m) error (Table 2). All three methods based on lap counting show a considerably lower IQR and CV for error, relative to the other two methods (Figure 5D). All the methods led to an underestimation of the distance compared to the measurement (D_ref_) with markings on the track. Results of the Bland-Altman analysis are provided in the Supplementary Figures S1–S5. Estimation of the MAS and sVT2 using the D_ref_ as predictor metric in linear regression led to R^2^ values of 0.93 and 0.93, respectively, and RMSE of 0.91 and 0.88 kmh^−1^, respectively. We obtained following linear equations:


(4) $$\begin{array}{r} \text{MAS}=5.0629\;\times \;{\text{D}}_{\text{ref}}+1.5427 \end{array}$$

(5) $$\begin{array}{r} \text{sVT}2=4.6486\;\times \;{\text{D}}_{\text{ref}}+0.7878 \end{array}$$

where MAS and sVT2 are in kmh^−1^ and D_ref_ in km. Bland-Altman analysis for the prediction of sVT2 using this equation is presented in the Supplementary Figure S6. Adding the biomechanical metrics to the D_ref_ as additional predictor metrics marginally improved the prediction, with R^2^ values of 0.93 and 0.93, and RMSE of 0.88 and 0.81 kmh^−1^, respectively, for MAS and sVT2.

**Table 2:** Error rates for the five distance estimation methods.

**Method**	**MAE (m)**	**MAE (%)**	**Bias (m)**	**CV (%)**	**LOA 1 (m)**	**LOA 2 (m)**
D_S_	250	8.9	−250	4.1	−30	−470
D_C_	102.7	3.4	−83	3.7	120	−290
D_L_	30.4	1.07	−17	1.2	49	−84
D_LS_	43.5	1.6	−36	1.3	38	−110
D_LC_	26.5	0.9	−16	1.1	44	−76

The mean absolute error (MAE) is by subtracting each estimated distance from the reference value. The bias, coefficient of variation (CV), and the limits of agreement (LOA) were obtained through Bland-Altman plots.

### Selection of metrics

**Figure 6:**
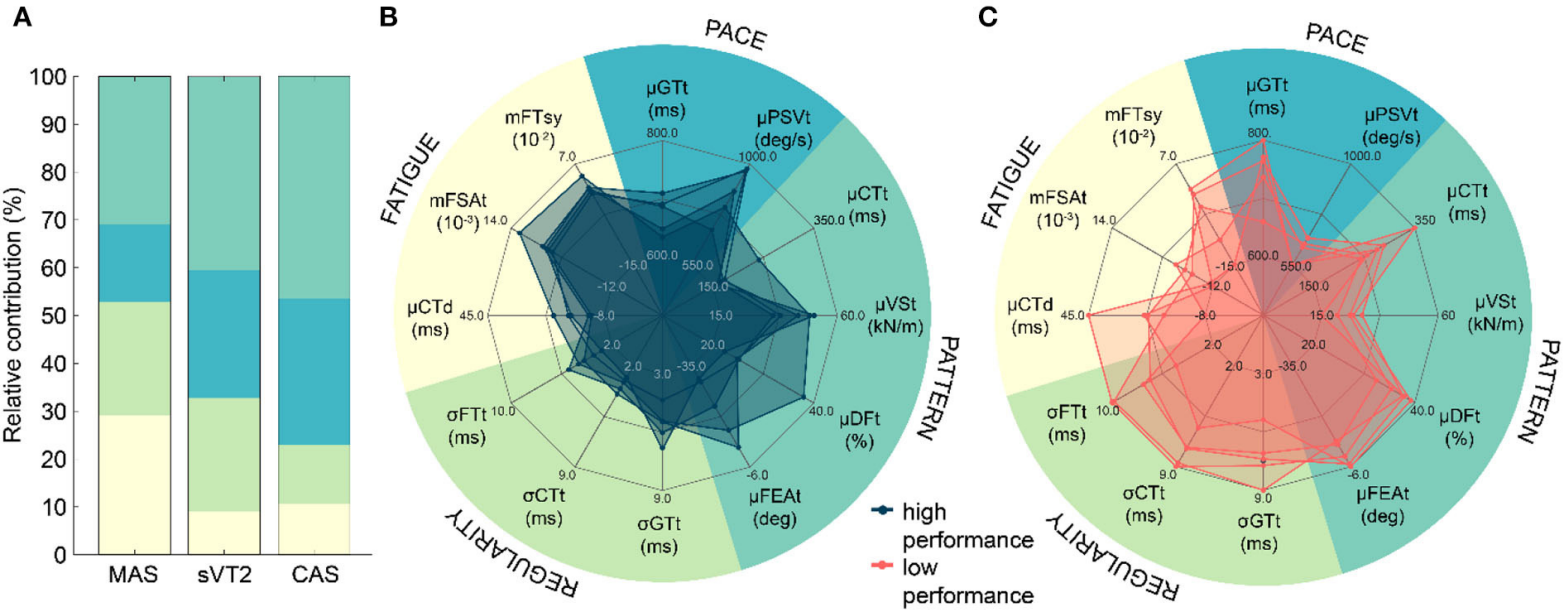
Selected metrics and their categories. **(A)** Relative contribution of metric categories to each endurance performance variable. **(B)** Biomechanical profile for top 5 (high performance) participants according to their MAS. **(C)** Biomechanical profile for bottom 5 (low performance) participants according to their MAS.

Using the method explained in Section Extraction of metrics and Table 1, we obtained a total of 175 biomechanical metrics for the 13 biomechanical parameters. Apart from SI parameters, the evolution of other parameters during the run is presented in Supplementary Figure S7. The number of metrics are reduced from 175 to 110 using multicollinearity, which were then used for statistical analysis and tested for correlation with the MAS, sVT2, and CAS. The final number of metrics for each performance variables were 33, 35, and 28, respectively. The cross-validated values for the fit of LASSO regression model for each performance variable are presented in Table 3. The model fits all variables with a R^2^ ≥ 0.65 and a RMSE of ≤1.80 kmh^−1^. The highest R^2^ and lowest RMSE is for the prediction of MAS. The biomechanical metrics selected through LASSO method for each performance variable are reported in Table 3, with a positive coefficient value indicating a positive contribution to the performance and *vice-verse* for negative values. The sum of coefficients for metrics belonging to the same category and their relative contribution is shown in Figure 6A. All performance metrics present a different relative contribution for each category. MAS shows a similar contribution for fatigue (29.2%) and technique (31%) categories, but sVT2 (40.4%) and CAS (46.5%) show a dominant contribution of the technique category. Exact value of the LASSO coefficients can be found in the Supplementary Table S2.

**Table 3:** Biomechanical metrics selected through LASSO regression and statistical testing.

**Performance variables**	**Fit quality**		**LASSO metrics**	
	**RMSE**	**R^2^**	**Positive contribution**	**Negative contribution**
MAS	1.62 kmh^−1^	0.75	μVSt, μPSVt, mSTt, mFSAt	σCTd, μCTt, σCTt, σGTt, σGTs, μCTd, mFTe, mFEAe, σDFt, μGTs, μFEAt
sVT2	1.78 kmh^−1^	0.65	μVSt, μPSVt, σFEAt, mVSe, mFSAt, mFTsy	σGTs, σGTt, μDFt, μGTs, σCTt, μFEAt
CAS	1.80 kmh^−1^	0.66	μVSt, μPSVt	μGTs, σCTt, μCTt
	**Pairwise statistical testing metrics**			
MAS	μCTt***, μGTt**, μVSt***, μFEAt***, μPSVt**, μDFt**, σCTt**, σFTt**, σFEAt*, mFSAt*, mFTsy**, μGTs**, μFSAs*, σCTs**, σFTs*, σGTs**, σFEAs*, mPSVs*, σCTe*, σFTe***, mFEAe*, μCTd*			
sVT2	μCTt***, μGTt**, μVSt***, μFSAt**, μFEAt**, μPSVt**, μDFt**, σCTt**, σFTt**, σFEAt*, mFSAt**, μGTs**, μFSAs***, σCTs**, σFTs*, σGTs**, σFSAs*, σFEAs*, mFSAs*, μFSAe*, σCTe*, σFTe**, mVSe*, μFSAd*, μCTd*			
CAS	μCTt***, μGTt***, μVSt***, μFEAt*, μPSVt***, μDFt**, σCTt**, σFTt**, mFSAt**, mFTsy**, μGTs***, σCTs**, σGTt*, mFSAs*, σCTe*, σFTe*, μCTd*			

Positive contribution denotes a positive coefficient obtained through the LASSO regression and vice-versa for negative contribution. Significant differences for pairwise statistical testing are indicated with

^*^p ϵ (0.01, 0.05),

^**^p ϵ (0.001, 0.01), and

^***^p ≤ 0.001.

Metrics showing a statistically significant (*p* < 0.05) difference between highest and lowest performing participants are also reported (Table 3). The effect sizes can be found in the Supplementary Table S3. MAS, sVT2, and CAS led to the selection of different biomechanical metrics, with the highest number of metrics selected for MAS through LASSO regression and for sVT2 through statistical testing. The metrics common to each performance variable across both methods were selected and used to create a biomechanical profile for the participants. The metrics included on the profile are (i) Technique: μCTt, μVSt, μDFt, μFEAt; (ii) Regularity: σCT, σFT, σGT; (iii) Asymmetry: none; (iv) Fatigue: μCTd, mFSAt, mFTsy; and (v) Pace: μGTt, μPSVt. Figures 6B,C show the profiles for the top and bottom 5 participants ranked according to their MAS, respectively.

## Discussion

In this work, we investigated the association between endurance performances quantified by three variables: MAS, sVT2, and CAS, and the biomechanical metrics measured during the performance of a Cooper test protocol. The selected metrics and the rationale behind their selection are discussed in this section. This is preceded by a short deliberation on the estimation accuracy of the distance ran during the test and the subsequent prediction of the three performance variables.

### Distance and speed estimation

Estimating the distance using all three lap counting methods led to better precision than the methods using strapdown integration of speed and Haversine formula alone. The lack of precision or the higher IQR of the error is likely due to the bias and the noise in the GNSS ground speed and latitude/longitude signal. The integration of the data from these signals leads to signal drift, which can vary considerably across participants, leading to a higher IQR of error. The GNSS ground speed is typically estimated using the phenomenon of Doppler shift while the Haversine formula relies on the actual co-ordinates recorded by the GNSS (Hofmann-Wellenhof et al., 2012), which could explain the differences between the errors for the two methods. The lap counting methods reduced the impact of drift by restricting the strapdown integration to signals recorded in partial laps.

Compared to the MAE for state-of-the-art GNSS sport watches (Gilgen-Ammann et al., 2020), the MAE for lap counting methods was similar or lower. However, the sport watches were tested for one participant, over a maximum distance of 4,296.9 m. While the GNSS sport watches underestimated the distance in urban and forest areas, they overestimated it on a running track. The authors (Gilgen-Ammann et al., 2020) attribute this overestimation under unobstructed conditions (Ranacher et al., 2016) to a possible correction algorithm used by manufacturers to compensate for the general underestimation in difficult areas. In our situation, we observed a general underestimation of distance by all five algorithms. One reason could be the lack of correction in the sensors, since they were used in the “airborne <4g” configuration of the uBlox GNSS chip. Another reason could be the assumption that all laps have a length of 400 m (Equation 3), which is lower than the actual distance for lane 2 (~407 m) and lane 3 (~415 m), which were used to compute the reference length during the Cooper test. The formula used in the lap counting algorithm can be easily updated to consider the lap length for a given lane (Aftalion and Martinon, 2019), thereby reducing the underestimation of distance.

MAS was estimated accurately (R^2^ 0.91, RMSE 0.98 kmh^−1^) with the Cooper test distance (D_ref_) as a sole predictor. This value of R^2^ is comparable to those in the literature for the prediction of VO_2max_−0.897 for the original study (Cooper, 1968), 0.87 to 0.93 for young males (Grant et al., 1995; McNaughton et al., 1998; Bandyopadhyay, 2015) and 0.72 to 0.83 in a systematic review (Mayorga-Vega et al., 2016) that determined the criterion validity of 12-minute Cooper test to be moderate for predicting VO_2max_. Although the addition of biomechanical metrics only improved the prediction slightly (R2 0.93, RMSE 0.88 kmh^−1^), it could prove to be more influential in case of studies with a larger and diverse set of participants. D_ref_ proved to be an accurate predictor of sVT2 (R^2^ 0.92, RMSE 0. 84 kmh^−1^) and addition of biomechanical metrics did not improve the prediction substantially (R^2^ 0.93, RMSE 0.81 kmh^−1^). To our knowledge, this is the first study to estimate sVT2 using the 12-min Cooper test. However, we recommend testing of this equation for a broader and larger set of participants. Estimation of sVT2 using a simple field test can enable its wider adoption for the design of threshold-based training programs and as a metric to measure the endurance capacity of athletes. Furthermore, estimation of sVT2 and MAS using field tests can facilitate studies which compare their predictive power for performance in endurance races and contrast their use in improving positive adaptation to training.

### Selection of metrics

The biomechanical metrics selected through LASSO for MAS, sVT2, and CAS differ from each other (Table 3). Similarly, participants in the high-/low-performance groups selected according to the highest and lowest MAS, sVT2, and CAS values differed, and consequently, the metrics showed statistically significant differences. These results highlight the dissimilarity of the nature of information obtained from these variables, although they all quantify the endurance performance. For the same fraction of VO_2max_ arising out of training at a certain fraction of MAS, athletes may have different levels of lactate accumulation, and therefore training based on fraction sVT2 can lead to a more homogenous training stimulus (Mann et al., 2013). Both, MAS and sVT2 can be reliably and accurately estimated using D_ref_ (or CAS), as shown previously. However, D_ref_ (or CAS) also contains information about the efficient conversion of endurance capacity on the track, which is determined by the running biomechanics and the running economy (RE). One study has shown that the high aerobic capacity of Kenyan runners is not reflected in treadmill running, due to their lack of familiarity and the resulting negative influence on RE (Saltin et al., 1995). Our results highlight the importance of running technique, with the ‘technique' category making the highest relative contribution to the estimation of CAS (Figure 6A).

The metrics selected within ‘technique' category are: μFEAt, μCTt, μVSt, and μDFt. Mean foot eversion angle (μFEAt) had a negative contribution to MAS and sVT2, as indicated by the LASSO coefficients (β) ranging from −0.26 to −0.02, with the faster runners having a higher inversion angle at initial contact. This result is consistent with previous studies that reported that an increase in running speed resulted in an increase in the ankle roll angle and thus the amount of external rotation (Muñoz-Jimenez et al., 2015; Orendurff et al., 2018). Foot roll before contact is lower in athletes with heel-strike and increases with midfoot and frontfoot strikes (Lieberman et al., 2010), leading to a higher inversion angle at contact. Midfoot strike loads the calf and shin muscles similarly, thereby stabilizing the ankle; forefoot strike causes the outer part of the foot to strike the ground at contact, preloading the calf muscles and allowing for a quick push-off with a minimal contact phase (Almeida et al., 2015). We observed a higher CT and FSA in slower runners, thus indicating a tendency toward heel-strike. This tendency, in combination with the lower speed, may explain the lower inversion angles observed in slower runners.

All three performance variables were negatively related [β ϵ (−0.39, −0.08)] (Table 3) to mean CT over 12 mins (μCTt). The five fastest runners had a lower μCTt than the five slowest (Figure 6). μCTt and gait cycle time are negatively affected by the gait speed and thus we might expect a lower μCTt for faster runners, regardless of their technique. However, a lower mean DF over 12 mins (μDFt) was also observed in the faster runners (Figure 6), and μDFt had a negative [β ϵ (−0.37, −0.24)] contribution (Table 3) to the performance variables. These findings highlight the fact that lower μCTt was due to running technique and not just the speed. Similar findings of lower μDFt and μCTt have been reported in treadmill running for the comparison between elite and highly trained runners (Burns et al., 2021) for a speed range (10–24 kmh^−1^) and a larger cohort of elite and well-trained runners at lower speeds of 10–12 kmh^−1^ (Folland et al., 2017). It has been reported that 10 km performance while running on an indoor track equipped with a force plate is moderately negatively correlated with CT (Williams and Cavanagh, 1987). Previous research has also linked a lower CT and DF to better performance in terms of RE (Nummela et al., 2007; Folland et al., 2017; Moore et al., 2019; Mooses et al., 2021).

In contrast to CT and DF, mean vertical stiffness (μVSt) contributed positively to all three performance variables [β ϵ (0.90, 1.2)], and the fastest runners had a considerably higher μVSt than the slowest runners (Figure 6). Similar results have been reported for comparisons between elite runners, well-trained runners, and other (non-runner) athletes during treadmill running (da Rosa et al., 2019; Moore et al., 2019; Burns et al., 2021). For a comparable propulsive force, a higher VS results in a lower vertical excursion of the center of mass (COM) and a lower mechanical energy loss due to vertical oscillations. The relatively lower CT and higher VS indicate the ability of faster runners to better utilize the spring mass dynamics for efficient storage and release of elastic energy during the stance phase (Zhang et al., 2021). With a rise in speed, the contribution of the elastic energy to the running energy cost has been shown to increase (Alexander, 1991), increasing the importance of efficient recycling of elastic energy. Ground reaction forces (GRF) have a strong positive influence on running speed (Weyand et al., 2000), but likely increase the vertical oscillation of COM, which is negatively correlated with RE (Saunders et al., 2004; Moore, 2016; Folland et al., 2017). Higher vertical and leg stiffness may reduce vertical oscillation while allowing for higher GRF, allowing higher speeds and better RE (Butler et al., 2003).

Within the ‘pace' category, two metrics were selected: μGTt and μPSVt. Mean gait cycle time (μGTt) had a negative [β ϵ (−0.45, −0.14)] contribution to the three performance variables, whereas mean PSV (μPSVt) had a positive [β ϵ (0.35, 0.72)] contribution. Faster runners had much lower μGTt and higher μPSVt compared with slower runners (Figure 6). For a given stride length, a lower μGTt results in higher running speed and is associated with higher vertical stiffness, which is consistent with our results (Butler et al., 2003). Even a 10% increase in step rate results in a considerable reduction in loading in the knee and hip joints, improvement in RE, and a reduction in vertical excursion of COM (Heiderscheit et al., 2011; Musgjerd et al., 2021; Quinn et al., 2021). An increase step rate results in more upright posture during stance, reducing the muscle forces needed during the loading-response phase of the gait cycle (Lenhart et al., 2014). Combining an increased step rate with a forefoot strike resulted in a greater reduction in joint impact loading than a midfoot or heel-strike (Huang et al., 2019). The transition to a forefoot strike at a higher step rate was also reported to be easier than midfoot and heel-strike in that order, which is consistent with our observation that faster runners report a lower μGTt and a tendency toward a midfoot and forefoot strike pattern. The lower μGTt increases the loading in the hip flexors muscles during the early swing because the trailing leg must be brought forward more quickly (Lenhart et al., 2014), possibly leading to an increased μPSVt. However, to decelerate the leg and position it for ground contact, the hamstrings and hip extensor muscles apply higher forces during the late swing phase. This indicates a higher capacity for positive and negative mechanical work in the thigh muscles for the faster runners.

The pace and technique categories primarily consider the mean values of the various biomechanical metrics. The acute fatigue developed during the Cooper test can affect the magnitude of the biomechanical parameters; so the fatigue category mainly considers the change in the mean values of the parameters. Within this category, three metrics were selected: μCTd, mFSAt, and mFTsy. Slower runners showed a higher increase in mean CT (μCTd) between the 2nd and 11th minute, indicating a limited ability to resist biomechanical changes due to fatigue. This is consistent with previous studies in which runners of different performance levels showed similar trends for the increase in CT with perceived acute fatigue (Prigent et al., 2022), but the magnitude of change in CT was higher in less-trained runners. In the fatigue category, the FSA and flight time (FT) are reduced less in the faster runners than the slow runners (Figure 6), leading to a higher slope for the FSA (mFSAt) and FT (mFTsy) in faster runners. This is reflected in the positive [β ϵ (0.07, 0.25)] contribution of mFSAt and mFTsy (β = 0.13) to the estimation of sVT2 and MAS. Acute fatigue may decrease calf muscle preactivation, resulting in a decreased ability to absorb and return energy generated during impact and produce a lower push-off force (Apte et al., 2021). Increased CT, to spread the impact impulse over a longer duration, a tendency of foot strike to move away from the forefoot (reduced FSA), and reduced FT indicate calf muscle fatigue, with less trained runners unable to adapt to these changes and recover their running technique.

The regularity category of metrics quantifies the variability of running and therefore the following metrics were selected within this category: σCT, σFT, and σGT. The variability of CT (σCT), FT (σFT), and GT (σGT) had a negative contribution [β ϵ (−0.47, −0.14)] to the estimation of all three performance variables. The fast runners showed a lower variability (Figure 6) of temporal gait parameters over 10-step windows, although they had lower mean values for these parameters. Gait variability has been previously studied with novice, well-trained, and elite runners on a treadmill (Nakayama et al., 2010; Mo and Chow, 2018; Burns et al., 2021), on a track (Meardon et al., 2011), and during a half-marathon (Apte et al., 2022a). With the exception of Meardon et al. who compared recently injured and healthy runners, all other studies found an inverse relationship between gait variability and training level. An increase in temporal gait variability was associated with an increase in energy cost of running (Candau et al., 1998). In a longitudinal endurance training program, a reduction in stride rate variability and an improvement in RE were reported as outcomes, although participants' oxygen capacity changed only slightly (Slawinski et al., 2001). Thus, the lower values of σCT, σFT, and σGT during the Cooper test indicate a better RE for the faster runners.

### Limitations and recommendations

The estimation of sVT2 in this study is based on a relatively small sample predominantly consisting of male subjects. The evaluation of the proposed equation (4) can be performed for a larger sample, with a better sex ratio, and possibly with nonlinear methods. Similarly, the well-trained runners were composed exclusively of male subjects, while the less-trained group was a mixture of male and female participants. The results of the comparison between the five fastest and the five slowest runners (Figure 6) are therefore biased by the low sex ratio. Some differences in the regularity of running mechanics occurred when competitive and recreational runners were compared within male and female subjects (Clermont et al., 2019a). However, males and females with similar training levels have been reported to have similar values for RE (mlO_2_ km^−1^ kg^−1^) (Daniels and Daniels, 1992) and the energy cost of running when running at a similar intensity (Bunc and Heller, 1989). In this study, the spring-mass model was used to estimate VS (Morin et al., 2005), based on the estimated values of FT and CT. Since VS showed the highest positive contribution for all performance variables, a direct estimation of VS using force plate measurements and motion tracking from COM may be a valuable follow-up study.

Reduction in the stability and smoothness of running movement, resulting from acute fatigue, has been linked to a surge in the energy cost of running (Schütte et al., 2018; Kiely et al., 2019). Using the IMU on the chest, it is possible to estimate the stability and smoothness of the trunk motion in real-world conditions (Apte et al., 2022b) and extend the proposed biomechanical profile. Together with the variability of gait temporal parameters, the long-range correlations (LRC) for stride time can be investigated, indicating the adaptability of gait. Highly trained runners and elite runners have shown a higher adaptability, and the LRCs have been associated with injury history (Meardon et al., 2011; Mo and Chow, 2018). However, the interpretation of the LRC, stability, and smoothness is not obvious for the coaches and the athletes; so we chose not to include these parameters. Finally, we relied on the pre-/post-measurement of the subjective fatigue (ROF) to ensure the maximal intensity for the Cooper test. Although the ROF scale correlates well with the biomechanical and physiological influences of acute fatigue (Prigent et al., 2022), it can be supplemented with a pre-/post-assessment of blood lactate. Finally, selected temporal metrics and μVSt can be investigated using a wrist-based IMU (Kammoun et al., 2022), enabling the use of smart watches for biomechanical assessment of the Cooper test.

## Conclusion

In this study, we presented an accurate (MAE 16.5 m) and precise (error CV 1.1%) estimate of the 12-min distance with a chest-worn GNSS receiver, despite interindividual variations in track running trajectories. Using this distance, we showed a reliable estimate [R^2^ > 0.9, RMSE ϵ (0.07, 0.25) kmh^−1^] of the MAS and sVT2, with reference values from the laboratory. Using the foot-worn IMU, we estimated a number of biomechanical metrics and assessed their contribution to the endurance performance. All performance variables were predicted with an acceptable error (R^2^ ≥ 0.65, RMSE ≤ 1.80 kmh^−1^) when only the biomechanical metrics were used with the LASSO method. The metrics selected using LASSO and the statistical comparison were used to create a biomechanical profile representing the running technique and its temporal evolution. Within this profile, the selected categories can be used to characterize runners and identify their key strengths and weaknesses. Based on this, a training program can be developed to target specific aspects of running technique and provide the resulting profile to runners as post-training feedback. This profile can be tracked over a season to understand the development of running technique and the adaptation of runners to training. Profiles at the beginning and the end of a long-distance training session reflect the impact of fatigue, providing complementary information to internal training load metrics. This profile can provide coaches and athletes a deeper insight into the running mechanics and allow evaluation of intraindividual changes following training programs and rehabilitation after injury. Interindividual differences in the profile can be used to develop a tailored training program and monitor the improvement in the resulting running mechanics. Use of such a wearable system in standardized capacity measurements may open a new perspective for personalization of training and rehabilitation.

## Data availability statement

The raw data supporting the conclusions of this article will be made available by the authors, without undue reservation.

## Ethics statement

The studies involving human participants were reviewed and approved by EPFL Human Research Ethics Committee (HREC 053-2020). The patients/participants provided their written informed consent to participate in this study.

## Author contributions

CB analyzed the laboratory sensor data. SA analyzed the field sensor data, conducted further data analysis, developed the linear models, and wrote the first draft of the manuscript. All authors conceptualized the study, with CB conducting the laboratory measurements and SA and ST conducting the field measurements. All authors contributed to the study design, discussion of the obtained data and results, and the final manuscript. All authors reviewed the final manuscript and assumed responsibility for the information presented therein.

## Funding

This project has received funding from the European Union's Horizon 2020 Research and Innovation Programme under the Marie Skłodowska-Curie (Grant Agreement No. 754354). Open access funding provided by École Polytechnique Fédérale de Lausanne.

## Conflict of interest

The authors declare that the research was conducted in the absence of any commercial or financial relationships that could be construed as a potential conflict of interest.

## Publisher's note

All claims expressed in this article are solely those of the authors and do not necessarily represent those of their affiliated organizations, or those of the publisher, the editors and the reviewers. Any product that may be evaluated in this article, or claim that may be made by its manufacturer, is not guaranteed or endorsed by the publisher.
