# Supplementary material for: Augmented Cooper test: Biomechanical contributions to endurance performance
Source: Front Sports Act Living. 2022 Sep 14;4:935272. doi: 10.3389/fspor.2022.935272 (PMC9515446; doi:10.3389/fspor.2022.935272)
Supplement: Supplementary file 1 [file Data_Sheet_1.pdf]

Supplementary Material

Table S1 Participant demographics and their performance according to the distance, MAS, and sVT2.

ID	Sex	Age	Height	Weight	MAS	sVT2	Distance	Speed
		years	cm	kg	km/h	km/h	m	km/hr
1	Male	40	181	77	18	15.4	3180	15.9
2	Male	25	182	73	19	16.4	3400	17
3	Male	31	179	64	19.5	17.1	3550	17.75
4	Male	30	167	51	20.25	18.7	3800	19
5	Male	22	173	55	21.5	18.9	3800	19
6	Male	30	185	66	21.5	19.3	3910	19.55
7	Male	26	182	67	19.50	16.6	3410	17.05
8	Male	25	187	82	18.75	16.2	3410	17.05
9	Male	28	184	83	19.5	17.3	3480	17.4
10	Male	41	177	73	19	15.4	3510	17.55
11	Male	25	178	69	18.5	15.9	3510	17.55
12	Male	25	175	80	19.50	14.4	3400	17
13	Male	19	179	57	19.50	18.5	3680	18.4
14	Male	25	173	54	19.5	17.1	3480	17.4
15	Male	25	178	84	20	18	3610	18.05
16	Male	24	183	75	19.5	17.7	3300	16.5
17	Female	30	162	49	15.5	13.2	2650	13.25
18	Male	34	180	65	14	11.6	2500	12.5
19	Male	34	176	73	17.25	13.8	2760	13.8
20	Male	31	175	63	12.5	10.3	2030	10.15
21	Male	31	175	66	17.5	15.8	3050	15.25
22	Male	27	180	77	17.5	16	3500	17.5
23	Female	33	156	45	11.25	9.3	1710	8.55
24	Male	39	187	95	10.5	10.3	1800	9
25	Female	25	168	51	11.5	12	2310	11.55
26	Male	30	183	76	16.5	13.7	2740	13.7
27	Female	21	160	56	12	10.4	2000	10
28	Male	47	183	79	14	12.3	2470	12.35
29	Male	28	184	80	15	14.2	2820	14.1
30	Female	28	158	50	14	13.3	2790	13.95
31	Male	29	175	78	16	13.7	2850	14.25
32	Male	29	178	87	12	10	2380	11.9
33	Male	35	180	70	13.5	10.9	2480	12.4

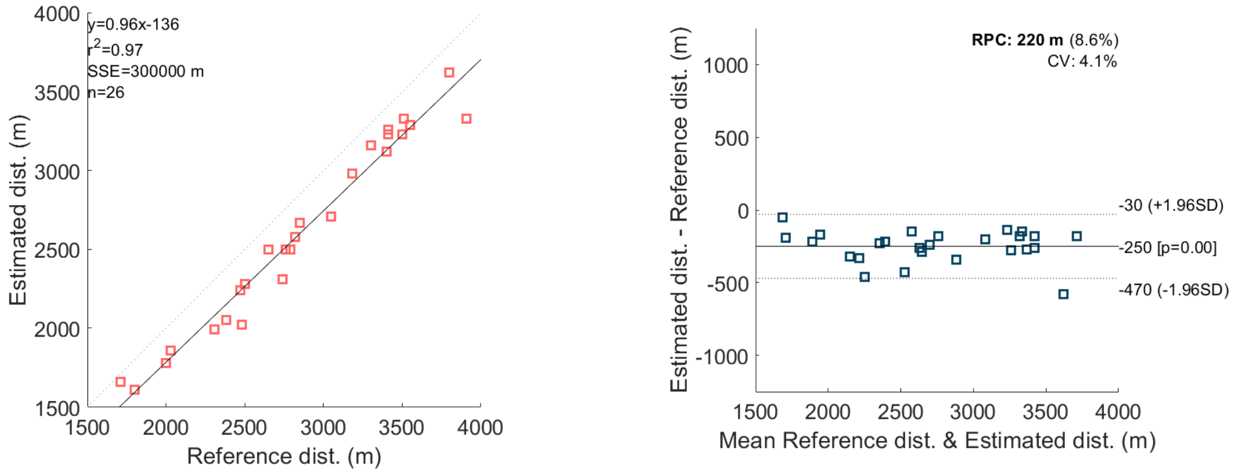

Figure S1 Bland-Altman analysis for distance estimation using method 1 (D_S)

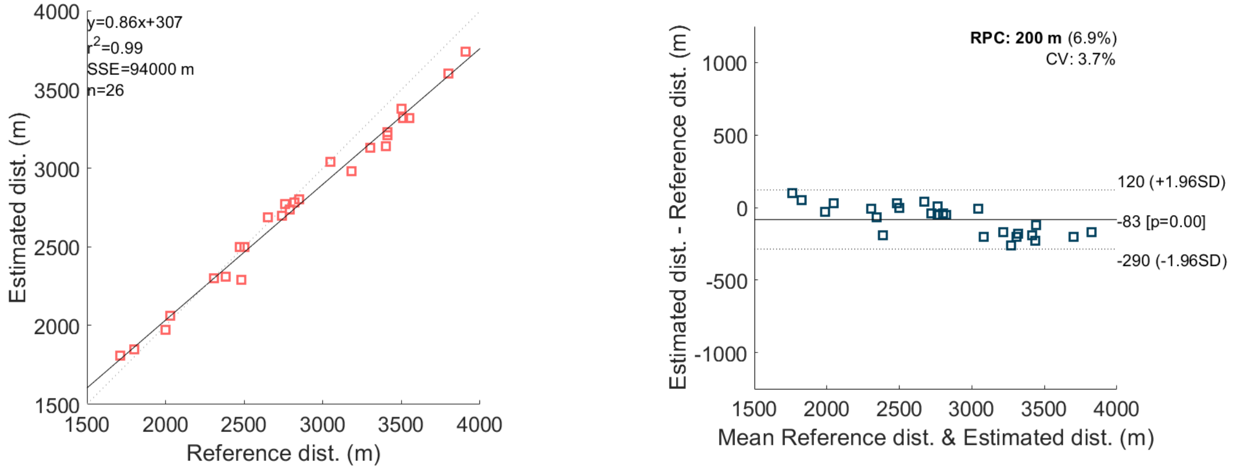

Figure S2 Bland-Altman analysis for distance estimation using method 2 (D_C)

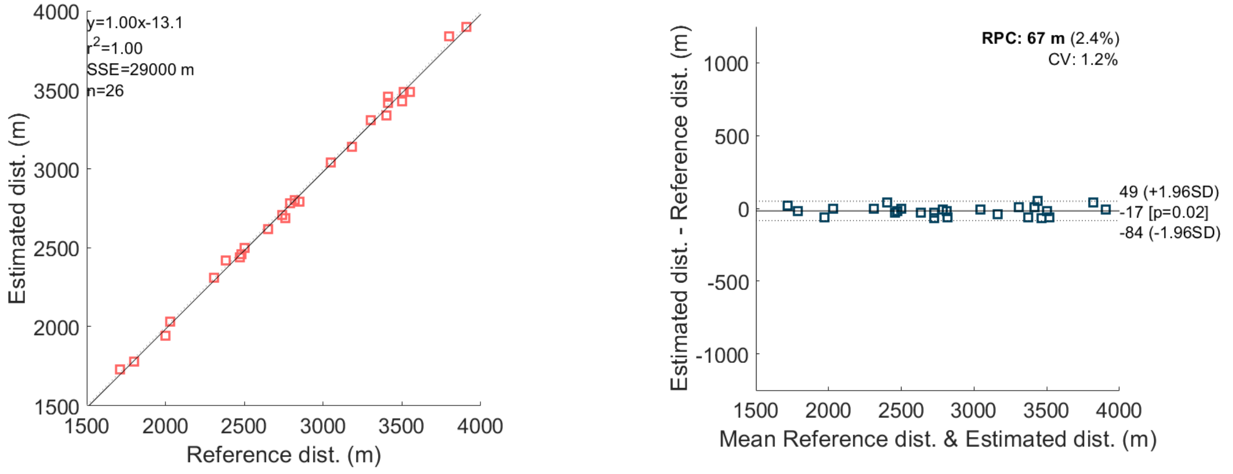

Figure S3 Bland-Altman analysis for distance estimation using method 3 (D_L)

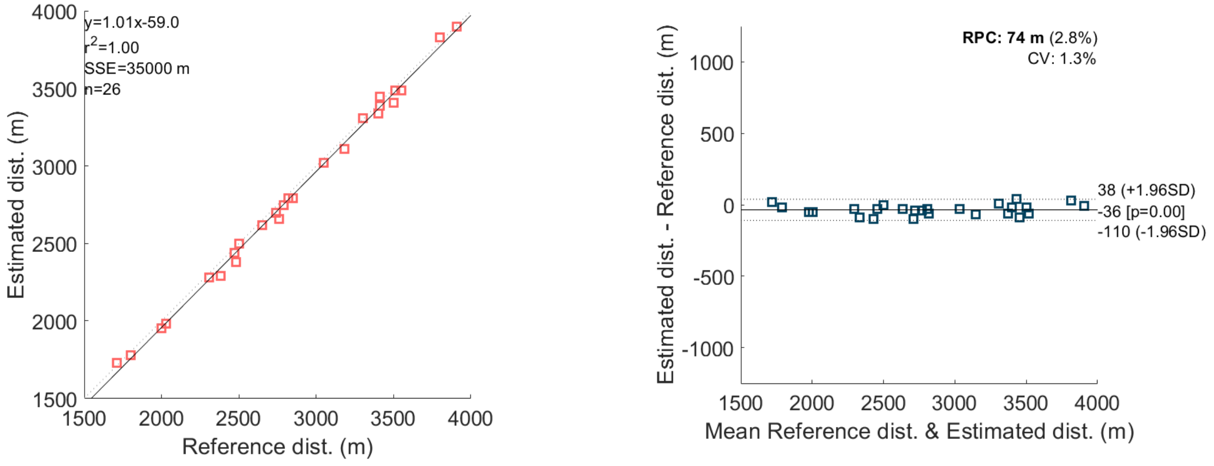

Figure S4 Bland-Altman analysis for distance estimation using method 4 (D_{LS})

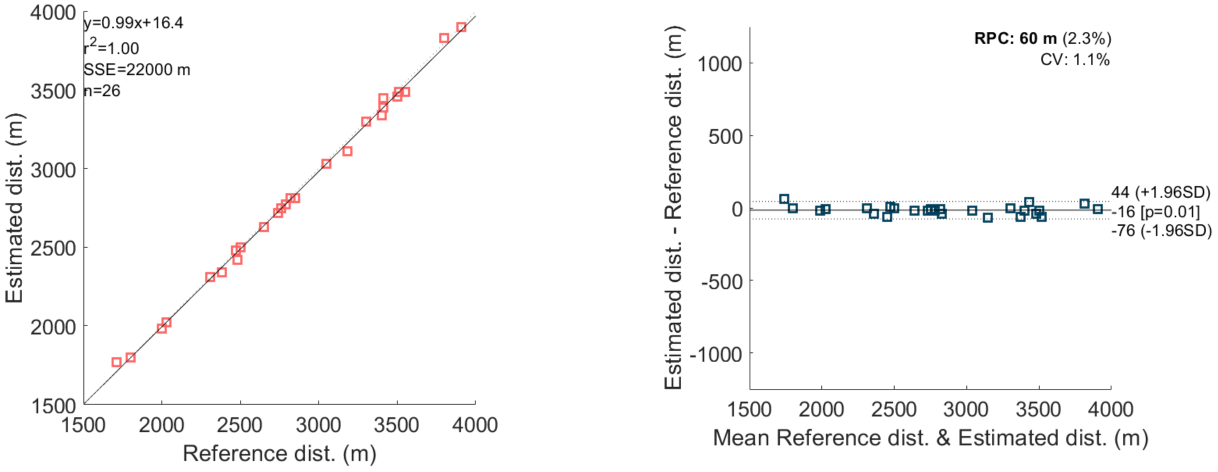

Figure S5 Bland-Altman analysis for distance estimation using method 5 (D_{LC})

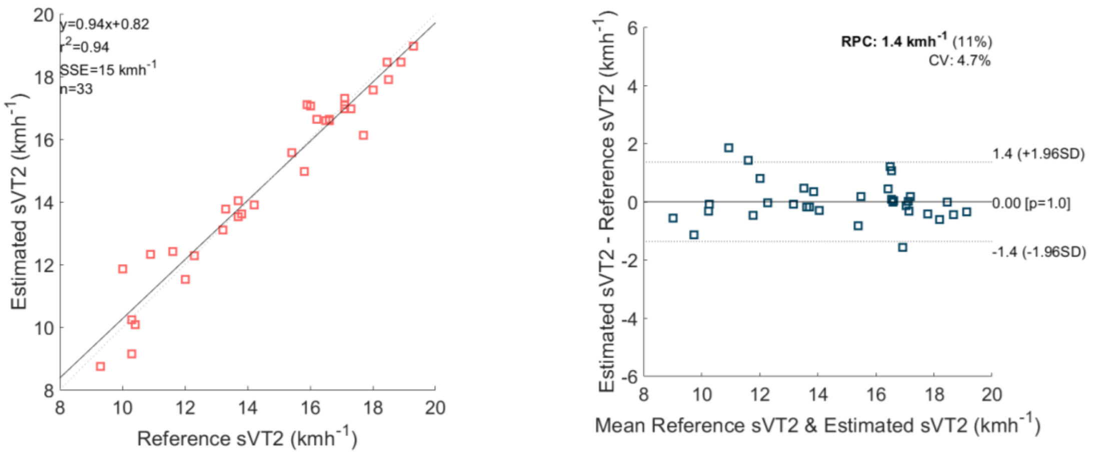

Figure S6 Bland-Altman analysis for sVT2 estimation using reference distance (D_{ref})

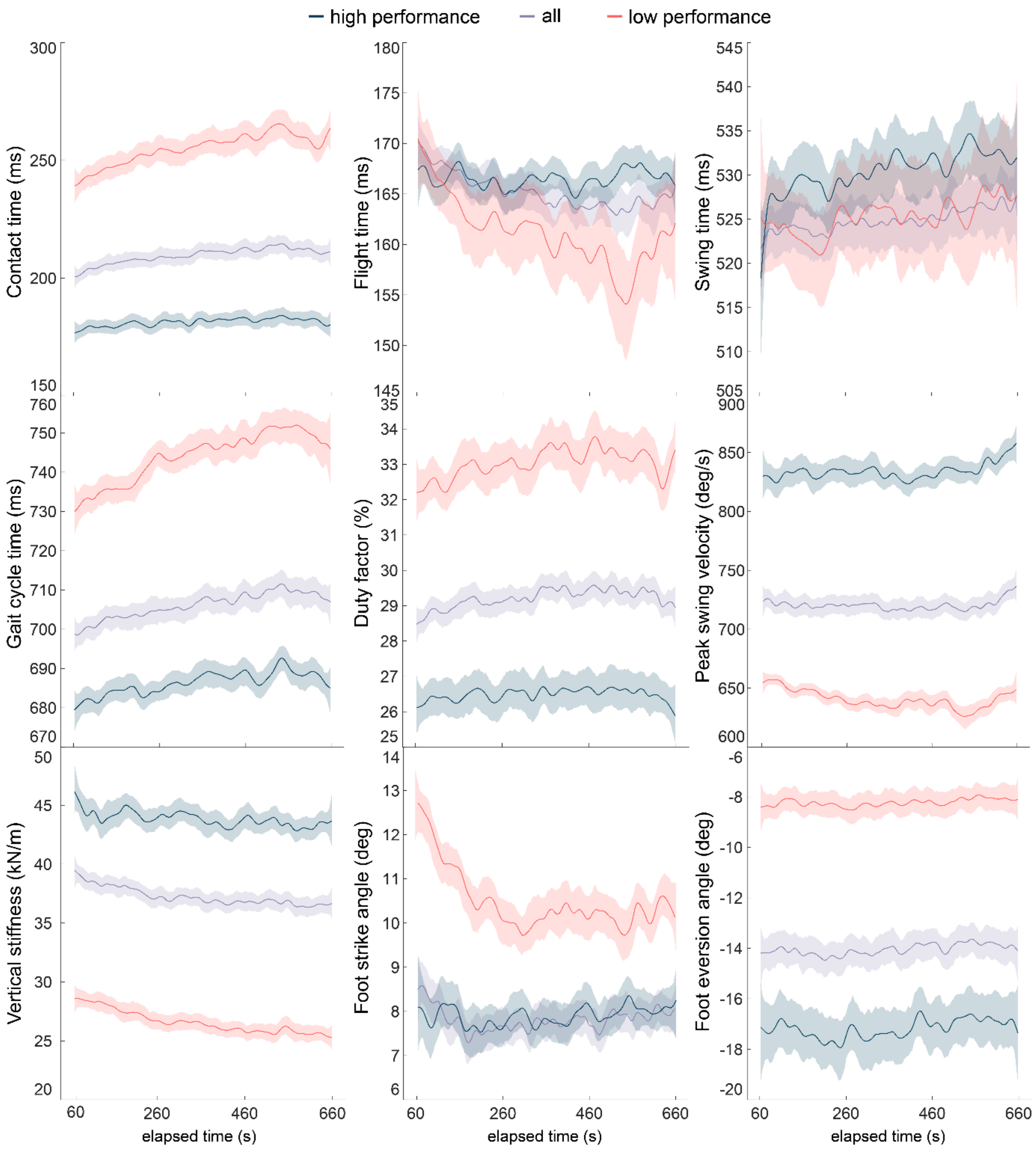

Figure S7 Profile for the biomechanical parameters during the Cooper test with participants grouped according to D_{ref}, The smoothed mean of original profiles and the 95% confidence interval is shown for easier comprehension of their overall group trend and and plotted using the Gramm toolbox (Morel, 2018). Compared to the high performance group, the low performance group typically showed a larger change in all the parameter values over 10 minutes, except for swing time and foot eversion angle at initial contact.

Table S2 Coefficients for the biomechanical metrics selected through LASSO. The three estimated performance variables were MAS (RMSE 1.62, R^2 0.75), sVT2 (RMSE 1.78, R^2 0.65), and CAS (RMSE 1.80, R^2 0.66).

MAS (kmh^{-1})		sVT2 (kmh^{-1})		CAS (kmh^{-1})	
Metric	Coefficient	Metric	Coefficient	Metric	Coefficient
µVSt	1.20	µVSt	1.08	µVSt	0.90
µPSVt	0.35	µPSVt	0.58	µPSVt	0.72
mSTt	0.30	σFEAt	0.16	µGTs	-0.14
mFSAt	0.25	mVSe	0.09	σCTt	-0.34
σCTd	-0.07	mFSAt	0.07	µCTt	-0.39
µCTt	-0.08	mFTsy	0.13		
σCTt	-0.20	σGTt	-0.14		
σGTt	-0.24	µDFt	-0.24		
σGTs	-0.25	µGTs	-0.37		
µCTd	-0.26	σCTt	-0.47		
mFTe	-0.26	σGTs	-0.07		
mFEAe	-0.26	µFEAt	-0.11		
σDFt	-0.31				
µGTs	-0.37				
µFEAt	-0.45				

Table S3 Biomechanical metrics that present a significant difference for pairwise statistical comparison.

MAS (kmh^{-1})			sVT2 (kmh^{-1})			CAS (kmh^{-1})		
Metric	p-value	ES	Metric	p-value	ES	Metric	p-value	ES
µCTt	0.0009	1.88	µCTt	0.0006	1.96	µCTt		1.95
µGTt	0.0032	1.52	µGTt	0.0026	1.56	µGTt		1.9
µVSt	0.00002	2.58	µVSt	0.00000	2.87	µVSt		2.63
µFEAt	0.0099	1.33	µFSAt	0.0059	1.4	µFEAt		1.29
µPSVt	0.00004	2.72	µFEAt	0.0041	1.58	µPSVt		2.75
µDFt	0.0072	1.36	µPSVt	0.0006	2.02	µDFt		1.33
σCTt	0.0013	1.72	µDFt	0.0062	1.39	σCTt		1.62
σFTt	0.0033	1.61	σCTt	0.0011	1.74	σFTt		1.42
σFEAt	0.0360	1.03	σFTt	0.0030	1.67	mFSAt		1.56
mFSAt	0.0231	1.14	σFEAt	0.0299	1.07	mFTsy		1.54
mFTsy	0.0081	1.34	mFSAt	0.0027	1.58	µGTs		1.76
µGTs	0.002	1.61	µGTs	0.0028	1.55	σCTs		1.47
µFSAs	0.0475	0.96	µFSAs	0.0009	1.8	σGTt		1.04
σCTs	0.0024	1.6	σCTs	0.0029	1.55	mFSAs		1.01
σFTs	0.0349	1.07	σFTs	0.0275	1.14	σCTe		1.02
σGTs	0.0041	1.48	σGTs	0.0022	1.61	σFTe		1.24
σFEAs	0.0362	1.03	σFSAs	0.0252	1.1	µCTd		1.02
mPSVs	0.0489	0.95	σFEAs	0.0341	1.04			
σCTe	0.0269	1.08	mFSAs	0.0239	1.15			
σFTe	0.0008	1.81	µFSAe	0.0179	1.17			
mFEAe	0.0284	1.1	σCTe	0.0226	1.12			
µCTd	0.0260	1.20	σFTe	0.0048	1.55			
			mVSe	0.0343	1.07			
			µFSAd	0.0448	1			
			µCTd	0.0283	1.14			
